# Intimate partner violence and child loss: an evaluation of 7 sub-Saharan African countries

**DOI:** 10.4314/ahs.v23i1.30

**Published:** 2023-03

**Authors:** Heather F McClintock, Sarah E Edmonds, Alexis R Lambert

**Affiliations:** 1 Department of Public Health, College of Health Sciences, Arcadia University, Glenside, PA; 2 Montgomery County Office of Public Health, Norristown, PA

**Keywords:** Partner violence, domestic violence, childhood mortality

## Abstract

**Purpose:**

Intimate partner violence (IPV) and child loss disproportionately affect women in sub-Saharan Africa (SSA). Little research has examined the relationship between IPV and child loss in SSA.

**Methods:**

We used data from Demographic Health Surveys in 7 countries in SSA (Côte d'Ivoire, Democratic Republic of the Congo, Namibia, Sierra Leone, Togo, Zambia, and Rwanda). Women's Health Module questions assessed lifetime physical, sexual, and emotional IPV. Child loss was calculated as the difference between the number of child births and the number of living children. Logistic regression was conducted adjusting for age, marital status, educational attainment, location of residence, wealth, sexually transmitted infections, and country of origin. Data were weighted and analysed using STATA Software (14.0).

**Results:**

Among women who gave birth, approximately one third (31.7%) reported that they lost 1 or more children. Nearly half (44.3%) reported that they experienced physical IPV during their lifetime. Women who had experienced physical, emotional, or sexual IPV were significantly more likely to report a loss of 1 or more children (OR=1.20, 95% confidence interval (CI)= [1.08, 1.33]; OR=1.30, 95% CI= [1.16, 1.45]; OR=1.42, 95% CI= [1.23, 1.65], respectively) in comparison with women who had not experienced IPV controlling for potentially influential covariates. Women who were older, married, had lower educational attainment, and had lower income were more likely to have lost 1 or more children.

**Conclusion:**

These results suggest that women who experienced all types of and cumulative exposure to IPV may be more likely to lose a child in SSA.

## Background

The United Nations Inter-Agency Group for Child Mortality Estimation reports that while notable progress has been achieved in reducing child mortality in sub-Saharan Africa (SSA), the region still reports the highest rates of under-five mortality in the world. In 2019, the average under-five mortality rate was 76 deaths per 1,000 live births. This rate is approximately 20 times greater than some developed regions and is substantially higher than the world's average child mortality rate. Projections indicate that by 2030 an additional 71 million youth and children may die globally, many of which will be in SSA. Hence there is a critical need for initiatives and interventions that aim to reduce the burden of child mortality in SSA.^1^

Social determinants of health are central in understanding the etiology of child mortality, and there is a growing need for research that assesses their impact. Intimate partner violence (IPV) is a social determinant that is widespread in SSA and can impact a mother's ability to foster child development. IPV refers to any behaviour within an intimate relationship that causes physical, psychological or sexual harm to those in the relationship.^2^ Experiencing one form of violence may double or triple the risk of experiencing additional forms of the violence.^3^ Hence, persons who experience IPV often experience multiple forms of IPV (also called cumulative exposure to IPV). Cumulative exposure to IPV may have a highly detrimental influence on physical, psychological, social, and economic indicators of well-being over the life course.^4^ Childhood is a critical period of development and cumulative exposure to IPV during this time can be harmful to many bio-psychosocial factors reducing the likelihood of child survival.^5^–^6^ The strong interconnection between different forms of violence is a growing area of scientific inquiry and is an important area of focus to expand knowledge on the etiology of IPV and child loss in SSA.

Countries in SSA often have a patriarchal structure in which gender roles have established men at the top of the hierarchy.^7^ Gender inequalities are associated with poor maternal and child health outcomes such as IPV.^8^–^9^ While men can experience IPV as well, the greatest known burden is experienced among women globally, this may be particularly notable in SSA where many women lack decision-making power in many facets of daily life.^10^ Some findings indicate that IPV may be prevalent across genders and sexual orientation.^11^ However, little research has assessed this relationship among transgender and gender nonconforming persons globally, constituting an important area of further research.

This is the first known study to evaluate the relationship between IPV and childhood mortality among multiple regions within sub-Saharan Africa (SSA). Prior work has shown that physical, emotional, and sexual IPV increases risk for child mortality.^12^–^14^ However, these previous investigations have primarily focused on single countries or regions (e.g. East) within Sub-Saharan Africa.^12^ A focus on SSA within multiple regions may have useful implications for targeted initiatives aimed at assessing, evaluating, and intervening across SSA (e.g. “Sisters for Life” microfinance and health education SSA program to address social factors and IPV).^15^ This work is also distinguished from previous research in that we examined cumulative exposure to violence. Many women who experience violence are victims of more than one form of IPV.^4^ Cumulative exposure to violence is an important predictor of well-being and outcomes providing useful information for intervention development and dissemination.^16^

In this study we used data from Demographic Health Surveys (DHS) to examine the relationship between IPV and child loss among women in SSA. This is the first known investigation to assess whether both the type of IPV and cumulative exposure to IPV may be related to child loss among women in SSA. The purpose of this study was to examine whether type of and cumulative exposure to IPV is associated with child loss in SSA. We hypothesized that women who were exposed to each type of violence as well as cumulative exposure to IPV would be more likely to have reported losing a child. By understanding whether IPV increases risk for child loss, we can inform the development of more effective initiatives that aim to reduce child mortality in SSA.

## Methods

### Sample and Procedures

Data for this study was obtained from 2011-2015 Demographic and Health Surveys (DHS) in seven sub-Saharan African countries (Côte d'Ivoire, Democratic Republic of the Congo, Namibia, Sierra Leone, Togo, Zambia, and Rwanda). These surveys are designed to be representative of the entire geographic area of interest. A sampling procedure was employed that was implemented in multiple stages. In the first stage geographical areas were identified using probabilities based on population size and relevant stratification demographic characteristics. Next, survey teams went to specific geographic areas that had been selected using systematic sampling. Using interview guides, household interviews were conducted with follow-up for persons eligible and willing to participate further. Prior work provides details on country specific sampling methodology.^17^ The domestic violence module is a part of the DHS Women's Module and contains questions focused on women's health as well as special measures to protect confidentiality. It contains IPV questions that were modified from the Conflict Tactics Scale (CTS).^18^ Survey interviewers who carried out the interviews received specialized training to protect participant safety and adhere to Institutional Review Board (IRB) requirements.^19^ Arcadia University Institutional Review Board deemed this study exempt.

### Measures

Dependent variable. Child loss was the outcome variable examined in this study. Child loss was calculated as the difference between the number of child births and the number of living children. This approach has been employed in prior work.^20^

Independent variable. Intimate partner violence (IPV) (physical, sexual, or emotional) by a current or former husband or intimate partner was measured by assessing 15 specific acts of violence. This aids in avoiding cultural differences related to violence. For each type of IPV a binary variable was created (yes/no) to assess the presence or absence of violence.

Cumulative exposure to violence was assessed by the number of different types (physical, sexual, and emotional) of violence that women reported that they had experienced. There were two main ways of categorizing cumulative violence. The first approach created two groups: women who had never experienced violence or those who had experienced one or more types of violence. The second approach created three categories: women who have experienced zero, one, or two/three types of violence both approaches were chosen to examine whether there were differences in the influence of IPV on child mortality between experiencing one or more types of violence in contrast to more incremental increases in exposure to IPV (one or two/three types).

Covariates. In the DHS standard questions were used to obtain basic demographic information about women and their current or former partner. Women's age was assessed in five-year increments. A wealth index measured wealth as per five categories: poorest, poor, middle, wealthier, or wealthiest. Marital status was assessed by assignment as not married, married, living with a partner, widowed, divorced, or separated. Residence was examined by categorization in a rural or urban setting. Finally, respondents also reported on their own as well as their current or former partner's education. Education was categorized by completing primary school or less or more than primary schooling because prior work has demonstrated these educational categorizations are associated with determinants of and the burden of IPV.^21^

### Data Analysis

The analysis was conducted in stages. First, descriptive statistics were assessed using frequencies and cross-tabulations. Second, chi-squared tests were used for bi-variate comparisons of women who had and had not lost a child. Finally, multivariate logistic regression was used to examine the relationship between IPV and child loss. Separate models were run to assess IPV in relation to one or more types of violence, one type of IPV, two or three types of IPV, physical IPV, sexual IPV, and emotional IPV adjusting for potentially influential covariates. Weighting was applied to account for the stratified cluster sampling of the study design in the final models. Results from fully adjusted models were presented as odds ratios and 95% confidence intervals (CI). Data was analyzed using STATA software version 14.0.

## Results

### Sample Characteristics

Participants ranged in age from 15 to 49 years old (mean=31.99 years, standard deviation (SD)=8.04). Most respondents were married (73.44%), resided in a rural setting (65.15%), and had less than or a primary education (74.84%). Approximately a quarter of women's partners had less than or a primary school education (26.05%). One third of women had lost one or more children (31.66%). Close to half (44.31%) of women reported exposure to one or more types of IPV.

**Figure 1:**
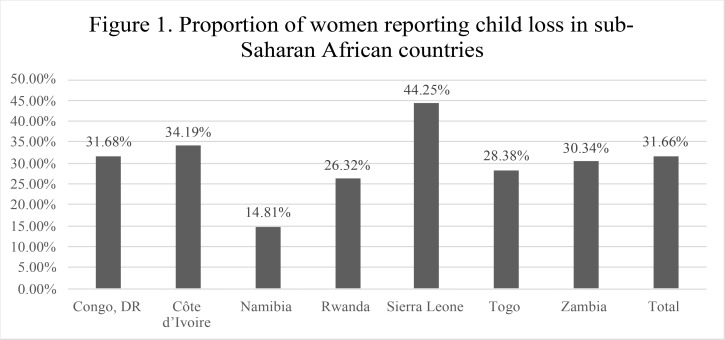
Proportion of women reporting child loss in sub-Saharan African countries

The proportion of women reporting loss of a child ranged from 14.81% in Namibia to 44.25% in Sierra Leone (Figure 1). The proportion of women indicating that they experienced one or more types of IPV ranged from 31.18% in the Ivory Coast to 56.65% in the Democratic Republic of the Congo. The proportion of women reporting IPV by country is shown in Table 1.

**Table 1:** Proportion of women reporting intimate partner violence and child loss by country (n=27,889)

Country	Any IPV (%)	1 type of IPV (%)	2 or 3 types of IPV (%)	Physical IPV (%)	Sexual IPV (%)	Emotional IPV (%)	Child Loss (%)
Congo, DR	56.65	20.21	36.44	46.29	25.58	37.05	31.68
Côte d'Ivoire	31.18	17.71	13.47	24.91	4.95	18.08	34.19
Namibia	34.98	16.18	18.80	25.53	7.35	25.95	14.81
Rwanda	40.42	17.88	22.54	31.92	11.36	27.26	26.32
Sierra Leone	49.08	23.87	25.21	43.10	6.98	28.17	44.25
Togo	37.75	19.20	18.55	21.90	8.07	31.83	28.38
Zambia	47.81	22.91	24.90	38.64	17.45	25.20	30.34
Total	44.31	20.57	23.74	34.63	13.40	27.80	31.66

Note: IPV, intimate partner violence; Congo, DR, Democratic Republic of the Congo

### Child Loss and Intimate Partner Violence

A higher proportion of women reporting child loss in comparison with those who did not experience child loss, experienced physical IPV (38.20% versus 32.97%, p<.001), sexual IPV (15.14% versus 12.60%, p<.001), and emotional IPV (30.97% versus 26.33%, p<.001). Similarly, a higher proportion of women reporting child loss in comparison with those who did not experience child loss, experienced one or more types of IPV (48.73% versus 42.26%, p<.001), one type of IPV (22.04% versus 19.88%, p<.001), and two or three types of IPV (26.68% versus 22.38%, p<.001). The relationship between socio-demographic characteristics, child loss, and IPV is shown in Table 2.

**Table 2:** Sociodemographic and partner characteristics of women completing the Domestic Violence module of Demographic and Health Surveys conducted in Democratic Republic of the Congo, Côte d'Ivoire, Namibia, Rwanda, Sierra Leone, Togo, and Zambia between 2011–2015, as categorized by child loss (n=27,889)

	Child Loss	No Child Loss	P-value
**Physical IPV**			
Yes	38.20%	32.97%	0.000
**Sexual IPV**			
Yes	15.14%	12.60%	0.000
**Emotional IPV**			
Yes	30.97%	26.33%	0.000
**Any IPV**			
Yes	48.73%	42.26%	0.000
**1 type of IPV**			
Yes	22.04%	19.88%	0.000
**2 or 3 types of IPV**			
Yes	26.68%	22.38%	0.000
**Age (years)**			
15–19	1.10%	4.88%	0.031
20–24	7.00%	19.58%	
25–29	15.40%	25.94%	
30–34	21.23%	20.98%	
35–39	21.81%	14.18%	
40–44	17.43%	8.75%	
45–49	16.03%	5.70%	
**Wealth Index**			
Poorest	26.36%	21.64%	0.000
Poor	23.72%	20.15%	
Middle	22.95%	20.37%	
Wealthier	17.66%	20.05%	
Wealthiest	9.32%	17.78%	
**Marital Status**			
Not currently married	23.63%	27.92%	0.002
Married	76.37%	72.08%	
**Residence**			
Rural	71.99%	61.98%	0.000
Urban	28.01%	38.02%	
**Education**			
Less than or Primary	84.79%	70.23%	0.000
More than Primary	15.21%	29.77%	
**Partner's education**			
Less than or Primary	34.06%	22.36%	0.000
More than Primary	65.94%	77.64%	

Note: IPV, intimate partner violence.

The results from fully adjusted models of the relationship between IPV and child loss are shown in Table 3. Women who experienced one or more types of IPV, one type of IPV, or two or three types of IPV were significantly more likely to have experienced child loss (AOR=1.32, CI=1.19, 1.45; AOR=1.23, 95% CI=1.08, 1.39; AOR=1.39, 95% CI=1.23, 1.57, respectively). Women who experienced physical IPV (AOR=1.20, CI=1.08, 1.33), emotional IPV (AOR=1.30; CI=1.16, 1.45), or sexual IPV (AOR=1.42; CI=1.23, 1.65) were more likely to have experienced child loss.

**Table 3:** Adjusted odds ratios and 95% confidence intervals for the association between intimate partner violence and child loss

	Any type of IPV AOR (95% CI)	One type of IPV AOR (95% CI)	Two or three types of IPV AOR (95% CI)	Physical IPV AOR (95% CI)	Emotional IPV AOR (95% CI)	Sexual IPV AOR (95% CI)
**Child Loss**						
Yes	1.32 (1.19, 1.45)	1.23 (1.08, 1.39)	1.39 (1.23, 1.57)	1.20 (1.08, 1.33)	1.30 (1.16, 1.45)	1.42 (1.23, 1.65)
No	1.00	1.00	1.00	1.00	1.00	1.00

Note: IPV, intimate partner violence; AOR, adjusted odds ratio; CI, confidence interval.

AORs adjusted for age, wealth index, marital status, residence, education, country of origin, and partner's education.

### Socio-demographic Factors and Child Loss

Among women who lost a child in comparison with those who did not lose a child, a higher proportion of women were within older age categories (aged 30-49), categorized as having less wealth (poorest, poor or middle), married, lived in a rural setting, and identified as having lower educational attainment themselves and for their partner as well (primary school or less). In the final model adjusting for covariates, women or their partners who had higher educational attainment and wealth status (wealthiest) were less likely to have reported losing a child. Women who were older and married were more likely to have reported loss of a child. No significant relationships were identified between child loss in relation to residence setting and sexually transmitted infection in fully adjusted models.

## Discussion

The aim of this study was to investigate the relationship between Intimate Partner Violence (IPV) and child mortality among women in sub-Saharan African countries. Exposure to each type of IPV (sexual, physical, or emotional) as well as cumulative exposures to IPV significantly increased the risk of experiencing child loss. This is the first known study to analyze the relationship between type of and cumulative exposure to IPV across multiple regions in sub-Saharan Africa. Our findings add to a growing body of literature indicating that there is a relationship between IPV and child mortality. Our findings may inform interventions that aim to reduce the burden of childhood mortality by increasing knowledge about the relationship between type of and cumulative exposure to IPV on child loss.

Our finding that the prevalence of child loss and IPV was relatively high in sub-Saharan Africa (SSA) is consistent with prior research. Rates of mortality in SSA are substantially higher than in other parts of the world. For instance, in 2019, SSA had the highest under-five mortality rate in the world with an average rate of 76 deaths per 1,000 live births which resulted in 2.8 million deaths in 2019, accounting for over half (53%) of the burden of child deaths worldwide.^1^ Our finding that IPV is common among women in SSA is also consistent with prior work. A recent study found that the pooled prevalence of experiencing any IPV among the ever-married women was 41.3% in SSA which was directly linked to socioeconomic factors such as education level, financial status, and rural residential location. 22 This estimate did not account for underreporting that can occur with self-reporting studies of IPV leading to the conclusion that the actual percent of ever-partnered women in SSA who have experienced IPV is significantly higher.

The effect of social determinants of health, such as IPV, on the increasing child mortality rate remains under studied. We found that each type of IPV (sexual, physical, and emotional) increased the risk of losing a child, when adjusting for covariates. This is consistent with previous research showing that physical, emotional, and sexual IPV increases risk for child mortality. 12–14, 23 Much prior research has focused on physical and/or sexual IPV in relation to child mortality. 24–25 In this study, we found that emotional violence was associated with an increased risk of child loss. These findings demonstrate that while physical and sexual violence have an impact on child loss, interventions may also need to incorporate emotional violence to reduce the burden of child loss. Furthermore, prior research has primarily focused on the relationship of IPV and child mortality within a singular African country or geographic region. 12, 23, 25–26 Our findings have implications for regionwide initiatives in SSA that may play a role in reducing IPV and childhood mortality. Many international governing bodies and institutions allocate resources through regionally based initiatives and our findings may garner support for IPV related work from these initiatives and funding mechanisms. Furthermore, addressing IPV may require a multi-level approach in which macro and systemic level changes are essential to support single-country and micro-level interventions. This approach requires engagement by institutions, governments, and policy makers. Regional approaches and engagement can create solidarity and push collective action forward to create significant change at the macro-level.

In this work we also found that women who experienced cumulative exposures to IPV (more than one type of IPV) were at an increased risk of experiencing child loss. The magnitude of the effect increased as the number of types of IPV increased suggesting that cumulative exposure may positively correlate with increased risk of child loss. Prior research indicates that cumulative exposure to IPV is very common. After experiencing one type of violence the risk of victimization from other types of violence doubled or tripled.^12^, ^20^, ^23^, ^26^ Strong interconnections between different forms of violence have been identified in much prior work indicating that different types of violence share common risk and protective factors.^27^

Social, community, biological and other factors that increase risk for one form of IPV lead to greater likelihood of perpetration of other forms of IPV. Exposure to cumulative violence is associated with poor outcomes that extend over the life course and in multiple areas of functioning including social, financial, and emotional domains.^20^, ^26^

Mechanisms that shape the relationship between IPV and child mortality have been investigated in prior research. The mechanisms are social, emotional, and biological in nature. The bulk of the global burden of IPV is experienced by women. 12, 28 This is particularly true in Africa where women are often dependent on men both emotionally and economically due to cultural differences in expected gender roles. 12, 20 The societal patriarchy often fosters cultural norms that promote and facilitate violence. As a result, women who face IPV also disproportionately face health issues and problems as well as psychosocial and mental effects which can occur prenatally or postnatally.^12^, ^23^ Cultural norms may cause women to lack autonomy to make decisions related to their sexual and reproductive health.^20^ Prenatally, effects such as physical trauma on the abdomen and stress hormones can cause outcomes like ante-partum hemorrhage, prematurity, and low birth weight babies.^12^, ^23^, ^24^, ^28^–^29^ IPV can reduce the mother's ability to care for herself and for the child which may lead to poor child attention, child malnutrition and ultimately, increased mortality. A poor mental state can lead to poor breastfeeding, substance abuse, and poor use of maternal health services. 20, 23, 30 Furthermore, children may experience direct effects from exposure to IPV victimization which could cause injury, maltreatment, or death.^23^, ^24^, ^28^, ^31^ Cumulative exposure to more forms of IPV increases the likelihood of experiencing additional risk factors for child mortality thus hindering child survival. Further research is needed to fully examine how cumulative exposure increases risk for child mortality.

Our results indicate that cumulative exposure may be critical in elucidating the relationship between IPV and child loss, giving insight into the magnified health impact of experiencing multiple types of IPV. Violence prevention initiatives may need to acknowledge and address the connections between different types of violence to increase their impact on reducing violence. This comprehensive approach can improve implementation strategies and approaches in ways that can more effectively protect individuals, groups and communities from violence. Understanding whether cumulative effects of IPV impact the chance of child mortality, may aid in developing interventions that address the mechanisms that drive the relationship.

This study's findings must be interpreted in the context of several limitations. First, the study utilizes cross sectional data which created a limited temporal interpretation between IPV and child mortality. The data does not allow conclusions to be drawn on whether IPV came before or after the event of a child's death. Additionally, the data source, the Demographic Health Survey (DHS), is done retrospectively and may introduce recall bias in the participants' answers. Despite this, the comprehensive nature of the DHS data allowed for the examination of multiple sociodemographic variables as well as a diverse range of demographic regions. Finally, due to the sensitive nature of IPV, under reporting of experiences may influence findings. Although some research has shown that the organizational and methodological features of the DHS have been attributed to lower assessment rates of IPV than other samples, it is anticipated that this effect is marginal and would have had little impact on the findings.^32^ The DHS also utilizes an in-depth process during data collection to ensure the privacy and anonymity of participants.

## Conclusion

This study's findings suggest that Intimate Partner Violence (IPV) may influence child mortality among women in sub-Saharan Africa. Women who experienced emotional, physical, and/or sexual IPV as well as cumulative exposures to IPV were more likely to report having a child die even when adjusting for potentially influential covariates. In addressing the disproportionate burden of child mortality in sub-Saharan Africa, future studies should focus on analysing specific mechanisms that drive the relationship between IPV and child mortality. Further in-depth research is needed to understand how interventions can most effectively intervene to reduce the impact of IPV on child mortality. Initiatives that may be a focus of further evaluation include education of practitioners related to screening, resources, and treatment that may improve outcomes for persons experiencing IPV. The empowerment of women to have greater autonomy in decision-making processes may play an important role in reducing IPV and child mortality.

WHAT IS ALREADY KNOWN ON THIS SUBJECT:

† Prior research, primarily in single countries, has shown that physical, emotional, and sexual IPV increased risk for child mortality.

WHAT THIS STUDY ADDS:

† This is the first known investigation to assess whether both the type of (physical, emotional, and sexual) and cumulative exposure to IPV may be related to child loss among women in multiple regions within SSA.

† We found that in a large sample of women in multiple sub-Saharan African (SSA) countries, women who were exposed to each type of and cumulative intimate partner violence (IPV) were more likely to experience child loss.

## Data Availability

The data that support the findings of this study are available in Demographic Health Surveys at https://www.dhsprogram.com/Data/. Survey interviewers who carried out interviews received specialized training to protect participant safety and adhere to Institutional Review Board (IRB) Requirements. The affiliated Review Board deemed this study exempt. (19-01-15).
